# Surgical occlusion of C1 spinal dural arteriovenous fistula

**DOI:** 10.3171/2025.7.FOCVID2573

**Published:** 2025-10-01

**Authors:** Karl L. Sangwon, Eric A. Grin, James S. Ryoo, Eytan Raz, Ilya Laufer, Erez Nossek

**Affiliations:** Departments of 1 Neurosurgery and; 2 Radiology, NYU Grossman School of Medicine, New York, New York

**Keywords:** cervical spine, craniocervical junction, dural arteriovenous fistula, intraoperative video, spinal arteriovenous fistula, vascular malformation

## Abstract

Spinal dural arteriovenous fistulas (dAVFs) at the craniocervical junction are rare vascular lesions that can cause progressive myelopathy and paralysis. This video presents a 40-year-old male with a left C1 spinal dAVF, who experienced unsteadiness, dizziness, leg weakness, and intermittent facial numbness. Given the lesion’s symptomatology, the patient underwent a C1 laminectomy and midline suboccipital craniectomy for definitive obliteration. The authors describe key surgical techniques for fistula exposure and obliteration, with intraoperative angiographic confirmation. Long-term follow-up confirmed complete persistent occlusion on angiography and resolution of his symptoms. This case highlights surgical strategies for managing dAVFs at the craniocervical level.

The video can be found here: https://stream.cadmore.media/r10.3171/2025.7.FOCVID2573

## Transcript

### 0:25 Patient History and Presentation.

A 40-year-old male with a history of hypertension presented with dizziness, leg weakness, and occasional left-sided facial numbness. Ten months prior to presentation, he experienced an episode of dizziness and leg weakness, along with a bifrontal headache. He reported similar episodes in the past, with slight unsteadiness when ambulating.

### 0:46 Diagnostic Angiogram.

A formal cerebral angiogram prior to the operation documented a dural AV fistula associated with the V3–V4 segment of the left vertebral artery and the C1 nerve root.^1^ A vascular network was seen surrounding the proximal V4 vertebral artery with venous drainage to the mesencephalic veins as well as into the occipital sinus with venous congestion.^2^

Superselective injection to the odontoid arch and left hypoglossal artery did not show any arterial contribution to the fistula. Three-dimensional reconstruction demonstrated the exact location of the fistula.

### 1:23 Surgical Planning.

The patient was evaluated for surgical embolization of the vascular lesion. During the angiogram, an attempt to engage the catheter into the arterial feeder demonstrated close proximity to the vertebral artery, which did not allow for safe endovascular embolization.^3,4^ Therefore, the decision was made to proceed with surgical intervention. The patient proceeded with C1 laminectomy and midline suboccipital craniectomy for occlusion of dural arteriovenous fistula with intraoperative angiogram.^5^

### 1:50 Surgical Approach and Exposure.

The patient was brought to the hybrid operating room and radial access was obtained for intraoperative angiogram. Patient was placed in a prone position. After C1 laminectomy and midline suboccipital craniectomy, the dura was opened approximately 2.5 cm in a paramedian fashion to avoid the occipital sinus, which was one of the draining systems of the fistula. The dura was tacked up using 2-0 silk sutures. The arachnoid was opened with an arachnoid knife and attached using hemoclips to the dura.

### 2:41 Identification of Structures.

The C1 nerve root and accessory nerve were identified, along with the vertebral artery. Distal venous outflow was noted. An arterial feeder taking off the dura and attached anterior to the vertebral artery was identified. ICG video angiography demonstrated the feeders and the main draining veins.

### 3:01 Obliteration of Fistula.

Further dissection revealed the main proximal draining vein between the dentate ligament and the dura. The main draining vein was followed to the level of its entrance into the dura. The most proximal venous outflow of fistulization was identified. A temporary clip was placed, and ICG video angiography demonstrated no flow within the distal draining vein.^6,7^

### 4:11 Intraoperative Angiogram.

A formal diagnostic cerebral angiogram confirmed complete obliteration of the fistulization.^8^

### 4:24 Confirmation of Obliteration.

The clip was removed, and the fistulization was obliterated using bipolar cauterization and separation with scissors. An additional ICG video angiography confirmed no flow into the draining vein.^7^ Irrigation was performed and hemostasis was achieved.

### 4:48 Closure.

The dura was closed with 6-0 Prolene in a running suture, and Gelfoam and Tisseel glue were placed on top of the dura. The muscle was closed with Vicryl sutures, and the skin was closed with 3-0 nylon. A drain was placed in the deep muscles, and a superficial JP (Jackson-Pratt) drain was placed. The patient was extubated and taken to the surgical ICU in stable condition.

### 5:28 Postoperative Course.

The patient’s postoperative course was uncomplicated. He experienced significant improvement in symptoms, with no recurrence of the previously noted C1 dural fistula. Neurologically, he was at baseline.

### 5:40 Follow-Up.

At the 1-year follow-up, angiography documented complete persistent occlusion of the previously treated fistula with no recurrence. The patient remained symptom free with no new complaints.

### 5:51 Conclusions.

This case demonstrates the successful surgical management of a complex C1 spinal dural arteriovenous fistula. The C1 laminectomy and midline suboccipital craniectomy approach, combined with meticulous dissection and intraoperative angiography, allowed for complete obliteration of the fistula with excellent clinical outcomes.

## Disclosures

Dr. Raz reported being a consultant for Balt, Imperative Care, Johnson & Johnson MedTech, Medtronic, Phenox, Q’Apel, Route92, Scientia, Siemens, Terumo Neuro, and Vasorum. Dr. Laufer reported personal fees from Globus and Icotec outside the submitted work. Dr. Nossek reported personal fees from Rapid Medical and Longeviti outside the submitted work.

## Author Contributions

Primary surgeon: Nossek. Assistant surgeon: Raz, Laufer. Editing and drafting the video and abstract: Nossek, Sangwon, Grin. Critically revising the work: Nossek, Sangwon, Grin, Ryoo, Raz. Reviewed submitted version of the work: Nossek, Sangwon, Grin, Ryoo, Raz. Approved the final version of the work on behalf of all authors: Nossek. Supervision: Nossek, Sangwon.

## Supplemental Information

### Patient Informed Consent

The necessary patient informed consent was obtained in this study.
